# Contribution of Prostaglandin Transporter OATP2A1/*SLCO2A1* to Placenta-to-Maternal Hormone Signaling and Labor Induction

**DOI:** 10.1016/j.isci.2020.101098

**Published:** 2020-04-27

**Authors:** Mai Inagaki, Tomohiro Nishimura, Takeo Nakanishi, Hiroaki Shimada, Saki Noguchi, Shin-ichi Akanuma, Masanori Tachikawa, Ken-ichi Hosoya, Ikumi Tamai, Emi Nakashima, Masatoshi Tomi

**Affiliations:** 1 Faculty of Pharmacy, Keio University, Minato-ku, Tokyo 105-8512, Japan; 2 Faculty of Pharmacy, Takasaki University of Health and Welfare, Takasaki, Gunma 370-0033, Japan; 3 Faculty of Pharmacy, Kindai University, Higashiosaka, Osaka 577-8502, Japan; 4 Graduate School of Medicine and Pharmaceutical Sciences, University of Toyama, Toyama 930-0194, Japan; 5 Graduate School of Biomedical Sciences, Tokushima University, Tokushima 770-8505, Japan; 6 Faculty of Pharmaceutical Sciences, Institute of Medical, Pharmaceutical and Health Sciences, Kanazawa University, Kanazawa, Ishikawa 920-1192, Japan

**Keywords:** Molecular Biology, Endocrinology

## Abstract

We evaluated the contribution of organic anion transporting polypeptide 2A1 (OATP2A1/*SLCO2A1*), a high-affinity carrier for prostaglandins (PGs), to the parturition process. At gestational day (GD) 15.5, OATP2A1 is co-localized with 15-hydroxy-PG dehydrogenase in the mouse placental junctional zone and facilitates PG degradation by delivering PGs to the cytoplasm. *Slco2a1* (+/−) females mated with *Slco2a1* (−/−) males frequently showed elevated circulating progesterone at GD18.5 and delayed parturition. Progesterone receptor inhibition by RU486 treatment at GD18.5 blocked the delay of parturition. In the junctional zone, PGE_2_ stimulated placental lactogen II (PL-II) production, resulting in higher expression of PL-II in *Slco2a1* (−/−) placenta at GD18.5. Indomethacin treatment at GD15.5 suppressed the PL-II overproduction at GD18.5 in *Slco2a1* (−/−) embryo-bearing dams, which promoted progesterone withdrawal and corrected the delayed parturition. These results suggest that extracellular PGE_2_ reduction by OATP2A1 at mid-pregnancy would be associated with progesterone withdrawal by suppressing PL-II production, triggering parturition onset.

## Highlights

• Placental OATP2A1 promotes PGE_2_ degradation by delivering PGE_2_ to the cytoplasm
• Placental PGE_2_ stimulates the PL-II production
• Extracellular PGE_2_ decrease by placental OATP2A1 promotes progesterone withdrawal
• Feto-placental OATP2A1 is associated with the initiation of parturition

## Graphical Abstract

**Figure:**
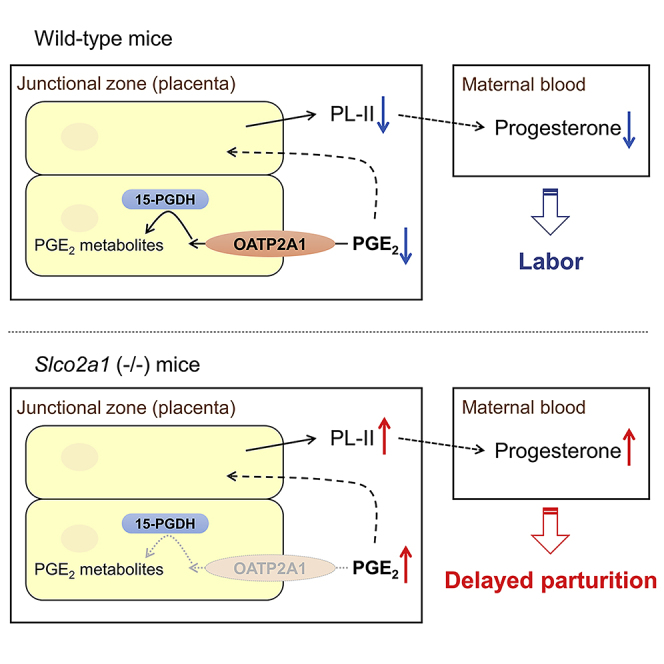

## Introduction

Pre-term (<37 weeks of gestation) and post-term (>42 weeks of gestation) deliveries are leading causes of prenatal death and morbidity worldwide (Liu et al., 2015, Saigal and Doyle, 2008, Challis et al., 2000, Minakami and Sato, 1996). Prostaglandins (PGs), especially PGF_2α_ and PGE_2_, have a uterotonic action in parturition and thus have been used clinically for induction of labor (Khan et al., 2008). In rodents, uterine PGF_2α_ production is induced via COX-1, and PGF_2α_ circulates to the ovary and binds to PGF_2α_ receptor to initiate corpus luteum regression, i.e., luteolysis (Sugimoto et al., 1997). Since progesterone is mainly produced by the corpus luteum, luteolysis reduces the circulating level of progesterone at late pregnancy (Malassine et al., 2003). Progesterone withdrawal, due to the decline of circulating progesterone levels, triggers COX-2 expression in the uterus, and COX-2-derived PGE_2_ and PGF_2α_ promote uterine myometrial contraction and cervical ripening (Tsuboi et al., 2003), resulting in induction of labor. Therefore, autocrine/paracrine signaling by PGs is an important determinant of the timely onset of labor.

*SLCO2A1*, encoding organic anion transporting polypeptide (OATP) 2A1, also known as PG transporter, is a high-affinity carrier for prostanoids (Kanai et al., 1995) and is thus capable of regulating autocrine/paracrine signaling by PGs. In the lung, OATP2A1 mediates the transport of extracellular PGE_2_ into alveolar epithelial cells, reducing extracellular PGE_2_ levels (Chang et al., 2010, Nakanishi et al., 2015). PGE_2_ *in utero* plays a critical role in maintaining patency of the fetal ductus arteriosus (Thorburn, 1992), and a fall in PGE_2_ level after birth triggers its closure (Coggins et al., 2002). In *Slco2a1*-deficient neonates, the ductus arteriosus fails to close after birth owing to the lack of pulmonary uptake of PGE_2_ (Chang et al., 2010). *In vitro* study has demonstrated that co-expression of OATP2A1 with 15-hydroxy-PG dehydrogenase (15-PGDH), a PG-degrading enzyme, accelerates the reduction of extracellular PG levels: PG inactivation involves active uptake into cells via OATP2A1 followed by cytoplasmic oxidation via 15-PGDH (Nomura et al., 2004). These findings indicate that OATP2A1 plays a central role in controlling extracellular PGE_2_ concentration and thus in signaling via the PGE_2_ receptor. In mice and humans, *SLCO2A1* mRNA is ubiquitously expressed across tissues but is most abundantly expressed in the placenta (Cheng et al., 2005, Kraft et al., 2010). The placenta has the ability to produce large amounts of PGE_2_ (Okazaki et al., 1981, Helliwell et al., 2006, Inagaki et al., 2017), and secretion of placental PGE_2_ into the fetal circulation has been proposed to maintain patency of the fetal ductus arteriosus (Thorburn, 1992). Nevertheless, the role of OATP2A1-mediated PG disposition in the placenta remains to be established.

In rat placenta, 15-PGDH is highly expressed in the junctional zone (Mark et al., 2013), which is positioned between the labyrinth and the maternal decidua. The junctional zone secretes placental lactogens (PLs) into the maternal circulation (Simmons et al., 2008). PL-I/*Prl3d* and PL-II/*Prl3b1* are considered to induce the production of progesterone in rodent luteal cells (Galosy and Talamantes, 1995, Thordarson et al., 1997, Zhong et al., 1997) and thus are proposed to be associated with the onset of parturition. This idea is supported by the fact that mice deficient in *Nrk*, a Ser/Thr kinase, show hyperproliferation of the junctional zone and delayed parturition of dams (Denda et al., 2011). Disruption of *Sirh7/Ldoc1*, a long terminal repeat retrotransposon, in mice likewise causes delayed parturition and overgrowth of the junctional zone with overproduction of PL-I (Naruse et al., 2014). It has been reported that the addition of PGE_2_ and PGF_2α_ increases corticotropin-releasing hormone secretion from cultured human placental cells (Petraglia et al., 1987). Thus, we hypothesized that, assuming OATP2A1 is localized in the junctional zone of the placenta, the manipulation of PG disposition by OATP2A1 affects placental endocrine function and thereby influences the timing of parturition.

Our aims in this work are to investigate the role of OATP2A1 in placental PG disposition and to evaluate the effect of *Slco2a1* deficiency on placental endocrine function and the timing of parturition. The upregulation of COX-1 expression in the uterus, triggering progesterone withdrawal occurs at gestational day (GD) 16.5 in C57/BL6 mice (Tsuboi et al., 2000), and therefore, in this study, we mainly analyzed phenotypes in *Slco2a1*-deficient pregnant mice at GD15.5 (mid-pregnancy), the day before progesterone withdrawal, and at GD18.5 (late pregnancy), the day prior to parturition.

## Results

### OATP2A1 Is Predominantly Expressed in Spongiotrophoblasts of Mouse Placenta

**Figure 1:**
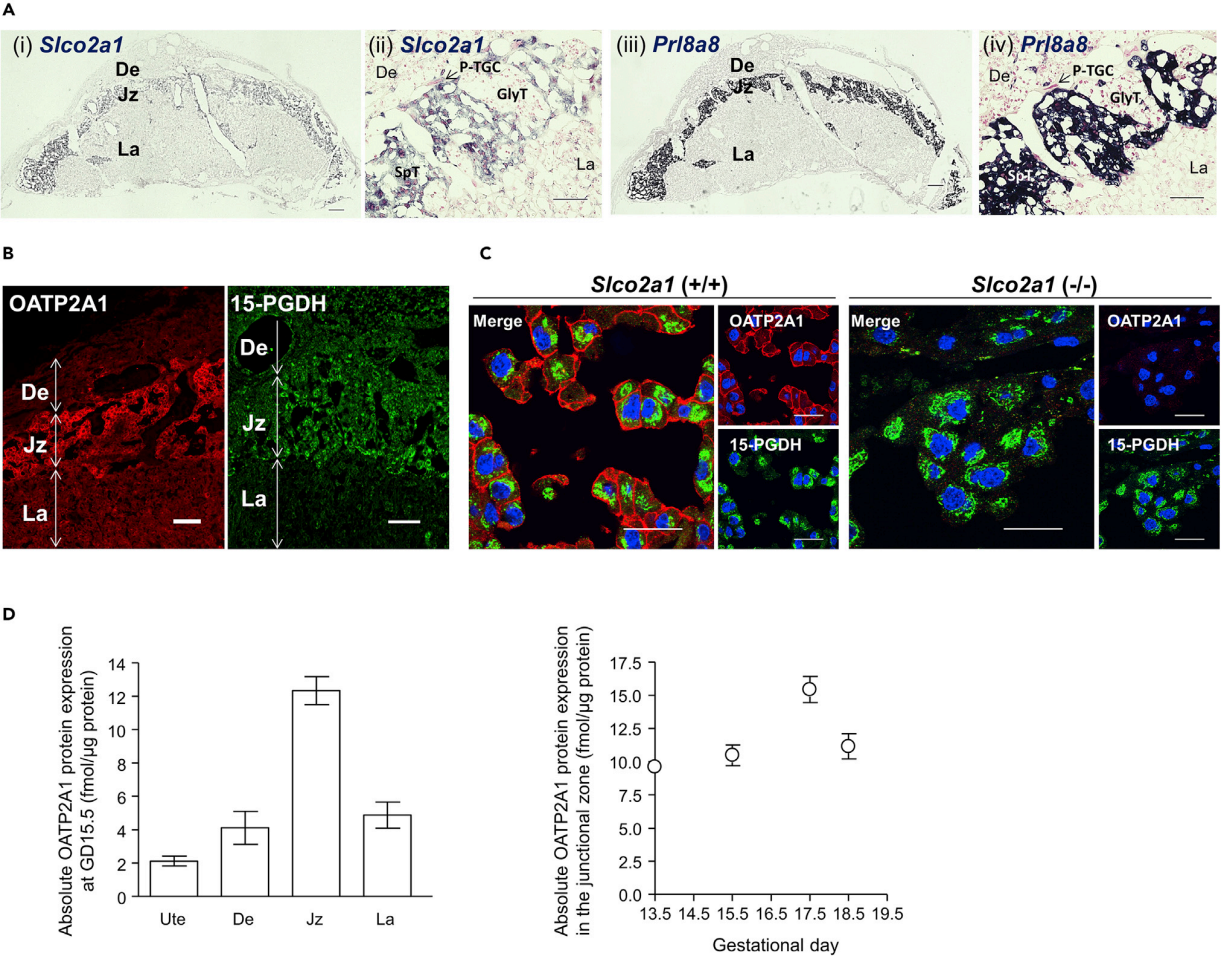
Placental Expression of OATP2A1 (A) *In situ* hybridization of *Slco2a1* (purple, i) and *Prl8a8* (purple, iii) in serial sections of GD15.5 mouse placenta. *Prl8a8* is used as a marker of spongiotrophoblasts. (ii) and (iv) are enlarged views of (i) and (iii), respectively. De, decidua; Jz, junctional zone; La, labyrinth; SpT, spongiotrophoblasts; GlyT, glycogen trophoblast cell; P-TGC, parietal trophoblast giant cell. Scale bars, 300 (i and iii) and 100 (ii and iv) μm. (B) Immunofluorescence of OATP2A1 (red) and 15-PGDH (green) in the mouse placenta at GD15.5. Scale bars, 120 μm. (C) Double immunofluorescence of OATP2A1 with 15-PGDH in the placental junctional zone of GD15.5 wild-type (left) and *Slco2a1* (−/−) placenta (right). Scale bars, 30 μm. (D) (Left) The absolute expression levels of mouse OATP2A1 protein in the plasma membrane-rich fraction. (Right) Gestational changes of OATP2A1 protein expression in plasma membrane-rich fraction of the junctional zone, from GD13.5 to GD18.5. Data are expressed as the mean ± SEM. Ute, uterus; De, decidua; Jz, junctional zone; La, labyrinth.

To investigate the distribution of *Slco2a1* mRNA in the placenta, sections obtained at GD 15.5 were hybridized with antisense RNA probe for *Slco2a1*. As shown in Figure 1A, *Slco2a1* is predominantly expressed in the junctional zone of fetal-derived placenta. In the junctional zone, which is composed of spongiotrophoblasts, glycogen trophoblast cells, and parietal trophoblast giant cells, staining of serial sections showed that the distribution of *Slco2a1*-positive cells (Figure 1A i and ii) coincides with the distribution of *Prl8a8* (Figure 1A iii and iv), a spongiotrophoblast marker (Simmons et al., 2008), strongly suggesting that *Slco2a1* mRNA is localized in spongiotrophoblast cells of the junctional zone. In addition, *Slco2a1* was weakly detected in parietal trophoblast giant cells (Figure 1A ii). Intense staining of OATP2A1 protein in the placenta at GD15.5 was selectively observed in the junctional zone (Figure 1B), in accordance with the distribution of *Slco2a1* mRNA (Figure 1A). Intense signals of 15-PGDH protein were detected in the maternal decidua and the junctional zone (Figure 1B). Double immunofluorescence staining showed that OATP2A1 (red) and 15-PGDH (green) were co-localized in spongiotrophoblasts of the junctional zone, and staining for OATP2A1 at the plasma membrane was diminished in placental sections prepared from *Slco2a1* (−/−) mice (Figure 1C). These results support the idea that 15-PGDH degrades PGE_2_ after it has been taken up into spongiotrophoblasts via OATP2A1. Placentas of *Slco2a1* (−/−) mice were morphologically indistinguishable from those of wild-type littermates at both GD15.5 and GD18.5 (Figure S1). Moreover, there was essentially no difference in fetal or placental weight between wild-type and *Slco2a1* (−/−) littermates (Figure S2).

We next quantified the absolute expression levels of OATP2A1 in the plasma membrane-enriched fractions from the uterus and three major zones of placenta (i.e., maternal decidua, junctional zone, and labyrinth) by detecting peaks of OATP2A1-derived peptides using LC-MS/MS. The absolute expression level of OATP2A1 protein in the junctional zone was more than 10 fmol/μg protein at GD15.5, which far exceeds that in any other tissue examined, and was consistently about 10 fmol/μg protein or more from GD13.5 to GD18.5 (Figure 1D). No signal peak was observed in the plasma membrane-enriched fraction from the junctional zone of *Slco2a1* (−/−) mouse placenta (Figure S3).

### OATP2A1 Is Involved in PG Degradation in Spongiotrophoblasts of Mouse Placenta

**Figure 2:**
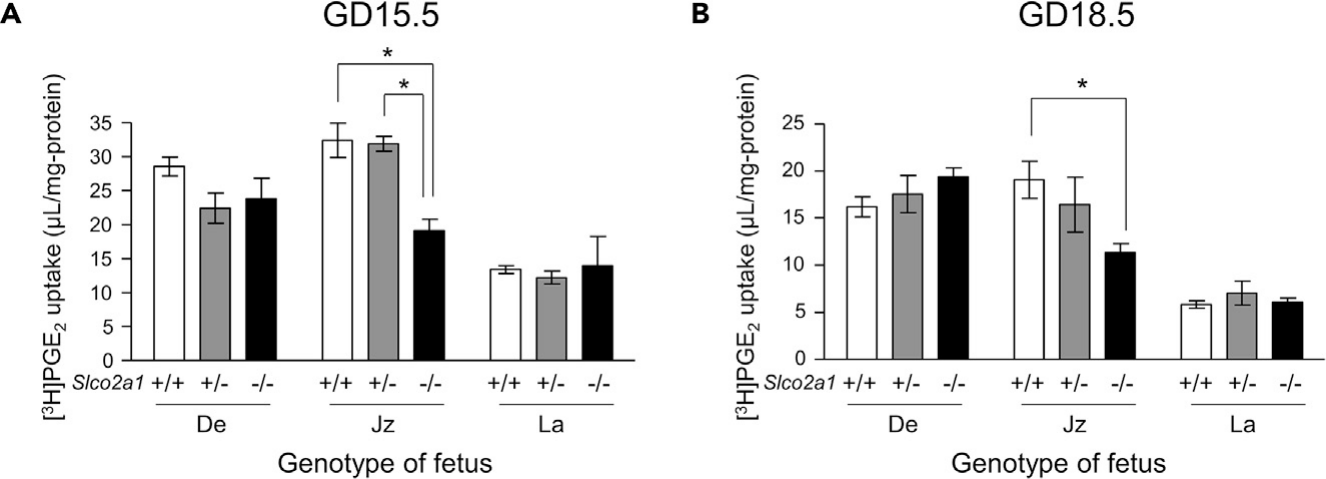
Effect of *Slco2a1* Deficiency on PGE_2_ Uptake by Placental Explants (A and B) Explants of the decidua (De), junctional zone (Jz), and labyrinth (La) at GD15.5 (A) and GD18.5 (B) were incubated for 20 min with [^3^H]PGE_2_ (5.6 nM) (n = 4–19). Data are expressed as the mean ± SEM. ∗p < 0.05, significant difference between groups (one-way ANOVA followed by Bonferroni's post hoc test).

To examine the function of OATP2A1 in the junctional zone, isolated explants of each placental zone at GD15.5 and GD18.5 were prepared for [^3^H]PGE_2_ uptake study. In the junctional zone of *Slco2a1* (−/−) mice, the uptake of [^3^H]PGE_2_ was significantly lower than that of wild-type littermates at both GD15.5 (Figure 2A) and GD18.5 (Figure 2B), indicating that OATP2A1 mediates the uptake of extracellular PGE_2_ into spongiotrophoblasts of the junctional zone. In the decidua and the labyrinth, there was no significant difference of [^3^H]PGE_2_ uptake between wild-type and *Slco2a1* (−/−) littermates (Figure 2).

**Figure 3:**
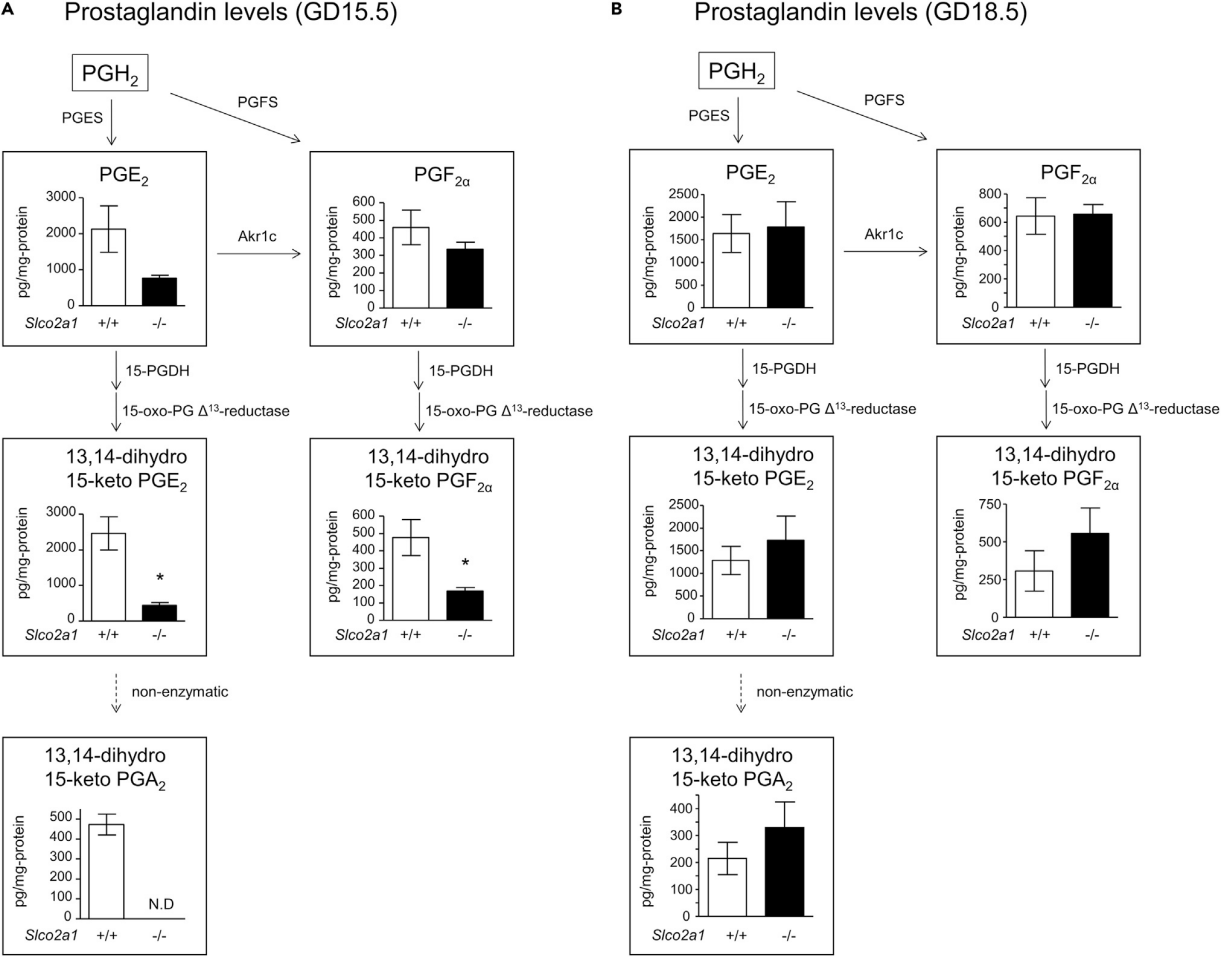
Effect of *Slco2a1* Deficiency on PG Levels in the Junctional Zone of *Slco2a1* (−/−) Mouse Placenta PG levels at GD15.5 (A) and GD18.5 (B) in the junctional zone, normalized to total protein, for wild-type (n = 5–6) and *Slco2a1* (−/−) placenta (n = 6–8) from *Slco2a1* (+/−) females mated with *Slco2a1* (+/−) males. Data are expressed as the mean ± SEM. ∗p < 0.05, significantly different between groups (Student's two-tailed t test). N.D., not detected.

We next measured endogenous concentrations of PGs in the homogenate of the junctional zone. PGE_2_ and PGF_2α_ levels in *Slco2a1* (−/−) mice junctional zone at GD15.5 tended to be lower; this may reflect a decline in intracellular concentrations owing to the lack of uptake via OATP2A1. There was a more marked effect on the concentrations of 13,14-dihydro-15-keto PGE_2_ and 13,14-dihydro-15-keto PGA_2_, which are stable metabolites of PGE_2_ generated by 15-PGDH (Figure 3A). Similarly, the level of 13,14-dihydro-15-keto PGF_2α_, a stable metabolite of PGF_2α_ generated by 15-PGDH, was also decreased in the junctional zone of *Slco2a1* (−/−) placenta (Figure 3A). These results suggest that the uptake of PGE_2_ and PGF_2α_ via OATP2A1 facilitates their degradation by 15-PGDH and consequently results in a decline of extracellular PGE_2_ and PGF_2α_ levels in the junctional zone. Conversely, the lack of PGE_2_ uptake via OATP2A1 appears to result in accumulation of extracellular PGE_2_. *Slco2a1* deficiency did not affect the expression levels of 15-PGDH- and PGE_2_-synthesizing enzymes (i.e., COX-1, COX-2, and mPGES-1) (Figure S4), and this further supports the idea that OATP2A1 itself largely caused the change in PGE_2_ level at GD15.5. In contrast to GD15.5, at GD18.5, there was no significant difference of endogenous PGE_2_ and PGF_2α_ metabolite levels between wild-type and *Slco2a1* (−/−) littermates in the junctional zone (Figure 3B), suggesting that extracellular accumulation of placental PGE_2_ and PGF_2α_ due to *Slco2a1* deficiency occurs at mid-pregnancy and not at late pregnancy.

### Feto-Placental OATP2A1 Is Required for Parturition

**Figure 4:**
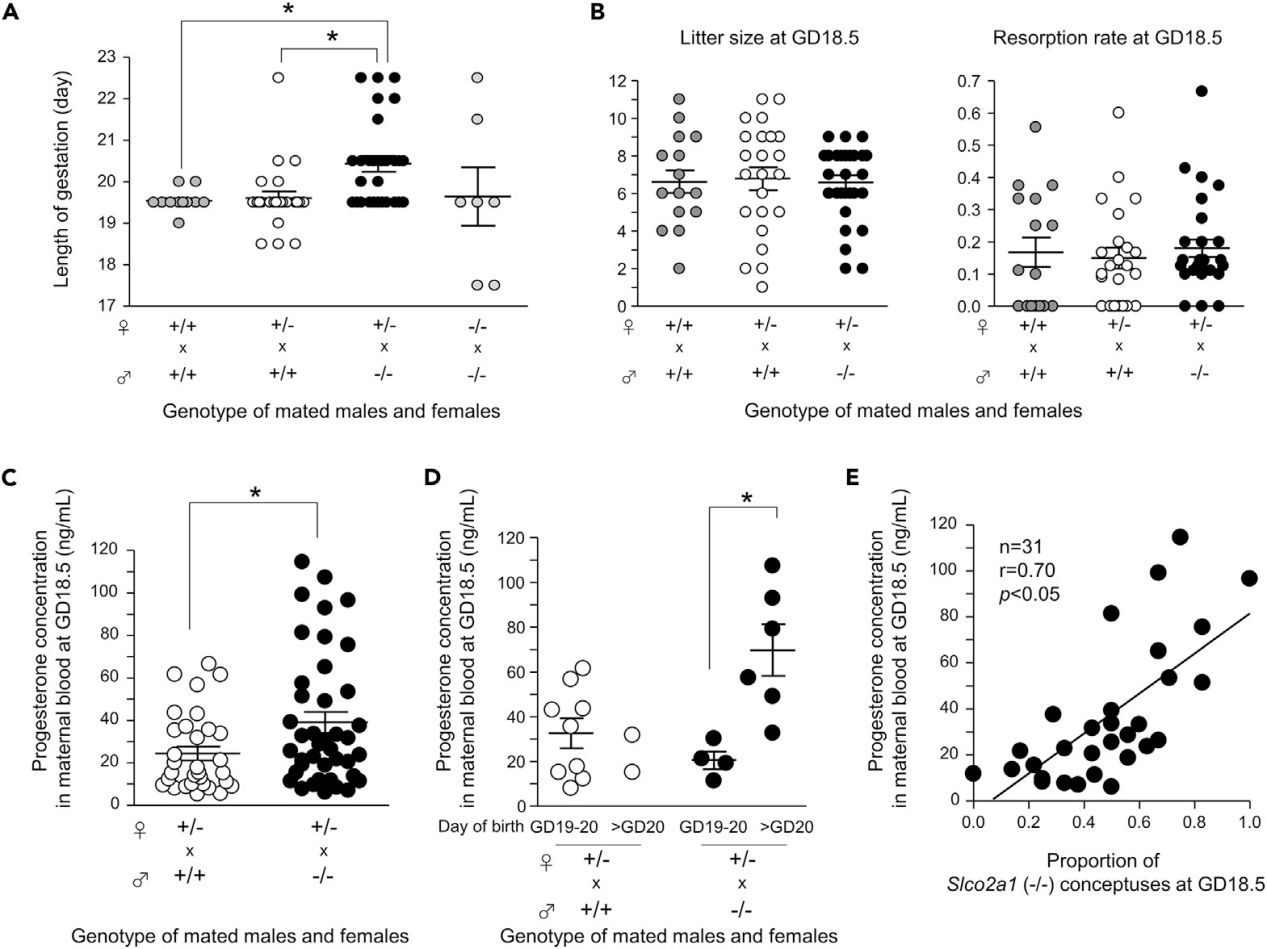
Prolonged Gestation of *Slco2a1* (+/−) Females Mated with *Slco2a1* (−/−) Males (A) Gestation length of wild-type females mated with wild-type males (n = 12), *Slco2a1* (+/−) females mated with wild-type males (n = 24) or *Slco2a1* (−/−) males (n = 29), and *Slco2a1* (−/−) females mated with *Slco2a1* (−/−) males (n = 7). (B) Number of pups (left) and resorption rate (right) at GD18.5 for wild-type females mated with wild-type males (n = 16), and *Slco2a1* (+/−) females mated with wild-type males (n = 17) or *Slco2a1* (−/−) males (n = 22). (C) Progesterone concentration in maternal blood at GD18.5 from *Slco2a1* (+/−) females mated with wild-type males (n = 31) or *Slco2a1* (−/−) males (n = 41). (D) Progesterone concentration in maternal blood at GD18.5 from *Slco2a1* (+/−) females showing term and post-term deliveries, mated with wild-type males (n = 11) or *Slco2a1* (−/−) males (n = 10). (E) Progesterone concentration in maternal blood at GD18.5 from *Slco2a1* (+/−) females mated with *Slco2a1* (−/−) males is positively correlated with the proportion of *Slco2a1* (−/−) conceptuses at GD18.5. Data are expressed as the mean ± SEM. ∗p < 0.05, significant difference between groups (A and B, one-way ANOVA followed by Bonferroni's post hoc test; C and D, Student's two-tailed t test; E, Pearson's correlation test).

In crosses of *Slco2a1* (−/−) mice, the mean length of gestation was comparable with that in wild-type counterparts, although 30% (2 of 7 cases) of dams exhibited post-term delivery (>GD20.0) and 30% (2 of 7 cases) of dams exhibited pre-term delivery (<GD19.0) (Figure 4A). To examine the requirement for placental OATP2A1 in parturition, we mated *Slco2a1* (+/−) females to wild-type or *Slco2a1* (−/−) males, giving rise to wild-type and *Slco2a1* (+/−) placentas/fetuses or *Slco2a1* (+/−) and *Slco2a1* (−/−) placentas/fetuses *in utero*, respectively. In crosses of *Slco2a1* (+/−) females to wild-type males, no difference in the timing of labor was evident compared with that in wild-type counterparts (GD19.6 ± 0.2; n = 24 versus GD19.5 ± 0.1; n = 12). Meanwhile, when *Slco2a1* (+/−) females were crossed with *Slco2a1* (−/−) males, more than 50% (16 of 29 cases) of dams gave birth at GD20.5 or later. Thus, a significant delay in parturition was observed in *Slco2a1* (+/−) females mated with *Slco2a1* (−/−) males (GD20.4 ± 0.2; n = 29, p < 0.05) compared with that in the females mated with wild-type males. These results indicate that the initiation of parturition is delayed by the high proportion of *Slco2a1* (−/−) conceptuses *in utero* (Figure 4A). There was no significant difference of litter size (6.6 ± 0.3; n = 33 versus 6.8 ± 0.5; n = 26) or resorption rate (0.16 ± 0.02; n = 33 versus 0.15 ± 0.03; n = 26) *in utero* at GD18.5 between *Slco2a1* (+/−) females mated with *Slco2a1* (−/−) males and those mated with wild-type males (Figure 4B). Even at parturition, there was no difference in pup number between these two crosses (5.7 ± 0.3; n = 27 versus 5.9 ± 0.6; n = 14). On the other hand, in crosses of *Slco2a1* (+/−) females to *Slco2a1* (−/−) males, the average number of weaned pups was significantly lower than that of the females mated to wild-type males (3.6 ± 0.2; n = 40 versus 6.7 ± 0.5; n = 22, p < 0.05), and the average proportion of *Slco2a1* (−/−) pups in a litter at weaning was significantly decreased compared with that at GD18.5 (0.10 ± 0.03; n = 40 versus 0.48 ± 0.04; n = 33, p < 0.05). These findings indicate that *Slco2a1* deficiency does not lead to embryonic lethality and that more than 70% of *Slco2a1* (−/−) pups die after birth and before weaning, presumably due to patent ductus arteriosus (Chang et al., 2010).

### Feto-Placental OATP2A1 Controls Progesterone Withdrawal

Progesterone is an absolute requirement for success of pregnancy, and withdrawal of progesterone signaling is critical for parturition (Cha et al., 2012). To examine whether progesterone withdrawal is impaired in *Slco2a1* (−/−) embryo-bearing dams, we measured circulating levels of maternal progesterone at GD18.5, when the circulating progesterone usually decreases in wild-type mice due to luteolysis. The progesterone levels in the maternal blood from *Slco2a1* (+/−) pregnancies produced by mating with *Slco2a1* (−/−) males were significantly higher than those produced by mating with wild-type males (Figure 4C). The expression level of *Akr1c18*, a progesterone-metabolizing enzyme, decreased to approximately half in *Slco2a1* (+/−) females mated with *Slco2a1* (−/−) males, compared with that in the females mated with wild-type males (data not shown). These findings strongly suggest that the circulating progesterone levels maintain high levels owing to a decline in the metabolizing enzyme expression in *Slco2a1* (+/−) females mated with *Slco2a1* (−/−) males.

In crosses of *Slco2a1* (+/−) females to *Slco2a1* (−/−) males, parturition was likely to be delayed (>GD20.0) in dams whose progesterone levels at GD18.5 had been high (Figure 4D). Moreover, when we plotted the proportion of *Slco2a1* (−/−) conceptuses for each of the crosses against circulating levels of maternal progesterone, we found that circulating progesterone levels at GD18.5 were positively correlated with the proportion of *Slco2a1* (−/−) conceptuses at GD18.5 in the corresponding litters (Figure 4E). These results suggest that impaired progesterone withdrawal is the major cause of delayed parturition in *Slco2a1* (+/−) females mated with *Slco2a1* (−/−) males. To verify this, we injected RU486, a progesterone receptor antagonist, into *Slco2a1* (+/−) dams bearing *Slco2a1* (−/−) embryos. Administration of 150 μg RU486 at GD18.5 invariably induced delivery of pups within 24 h (Table 1). In contrast, vehicle treatment of *Slco2a1* (+/−) dams bearing *Slco2a1* (−/−) embryos at GD18.5 frequently resulted in delayed parturition (Table 1). Overall, these findings suggest that feto-placental *Slco2a1* deficiency inhibits the drop in progesterone levels probably due to luteolysis and delays parturition.

**Table 1:** Effect of RU486 Administration on Parturition Timing in *Slco2a1* (−/−) Females Mated with *Slco2a1* (−/−) Males

Genotype of Mother	Genotype of Father	RU486 (μg)	Rate of Delivery within 24 h
*Slco2a1* (+/+)	*Slco2a1* (+/+)	150	100% (3/3)
*Slco2a1* (+/−)	*Slco2a1* (+/+)	150	100% (3/3)
*Slco2a1* (+/−)	*Slco2a1* (−/−)	0	43% (3/7)
*Slco2a1* (+/−)	*Slco2a1* (−/−)	150	100% (8/8)

RU486 was administered on GD18.5 to *Slco2a1* (+/−) females mated with wild-type or *Slco2a1* (−/−) males.

### Placental PG Regulates the Production of PL-II

**Figure 5:**
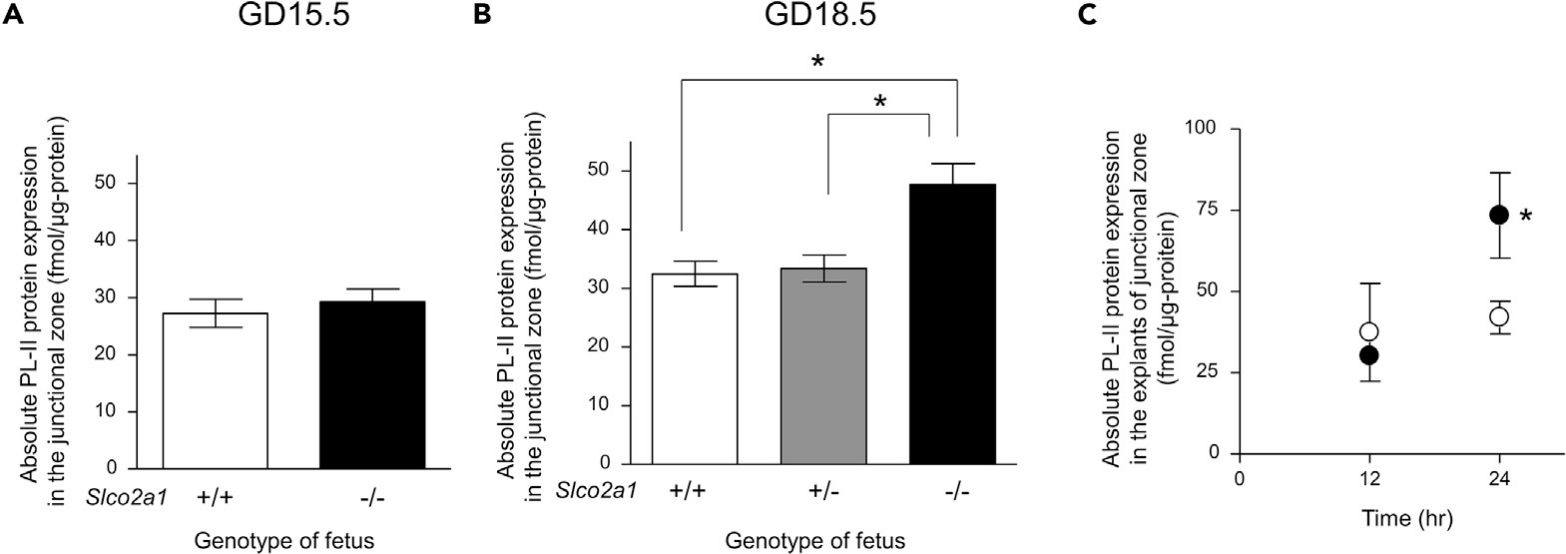
Overexpression of PL-II in the Junctional Zone of *Slco2a1* (−/−) Mouse Placenta (A and B) The absolute protein expression of PL-II at GD15.5 (A) and GD18.5 (B) in the junctional zone of wild-type (n = 9–13), *Slco2a1* (+/−) (n = 23), and *Slco2a1* (−/−) mice placenta (n = 9–10). (C) Absolute PL-II protein expression level in wild-type mouse explants of placental junctional zone cultured for 24 h in the presence (closed circle; n = 4–8) or absence (open circle; n = 4–8) of 1 μM PGE_2_. Data are expressed as the mean ± SEM. ∗p < 0.05, significant difference between groups (A and C, Student's two-tailed t test; B, one-way ANOVA followed by Bonferroni's post hoc test).

In order to examine the effect of *Slco2a1* deficiency on the expression levels of placental hormones, we measured the absolute protein expression levels of PL-I and PL-II in the junctional zone at GD15.5 (Figure 5A) and GD18.5 (Figure 5B). At GD15.5, there was no significant difference in PL-II expression between wild-type and *Slco2a1* (−/−) mice junctional zone. In contrast, at GD18.5, PL-II expression level in the junctional zone of *Slco2a1* (−/−) placenta was significantly higher than that of wild-type placenta (Figure 5B). The expression level of PL-I was under the limit of quantification at both GD15.5 and GD18.5 (data not shown).

In the junctional zone of *Slco2a1* (−/−) placenta, extracellular PGE_2_ appears to be increased owing to failure of PG degradation at GD15.5. Therefore, in order to examine the effect of PGE_2_ on PL-II expression, we cultured wild-type placental explants of the junctional zone in medium containing PGE_2_. PL-II expression in the junctional zone was significantly enhanced by PGE_2_ treatment when compared with the vehicle-stimulated control measured at 24 h (Figure 5C). Consequently, it is possible that failure of PGE_2_ degradation due to *Slco2a1* deficiency at mid-pregnancy resulted in the induction of PL-II expression at late pregnancy.

It has been suggested that pulmonary surfactant protein A (SP-A), secreted from matured fetal lung, serves as a fetal signal to initiate parturition in dams by inducing progesterone withdrawal (Gao et al., 2015). However, we found no significant difference of expression level of SP-A between wild-type and *Slco2a1* (−/−) fetal lung at GD18.5 (Figure S5). The levels of PGE_2_ and PGF_2α_ in uterus and ovary at GD18.5 were similar in *Slco2a1* (+/−) females mated with *Slco2a1* (−/−) males and in those mated with wild-type males (Figure S6). Accordingly, feto-placental OATP2A1 locally influences PGE_2_ and PGF_2α_ levels in the junctional zone of the placenta but does not affect those in maternal reproductive tissues, where PGs are involved in the induction of labor.

### Treatment with Indomethacin at GD15.5 Rescues the Delayed-Labor Phenotype in *Slco2a1* (+/−) Females Mated with *Slco2a1* (−/−) Males

**Figure 6:**
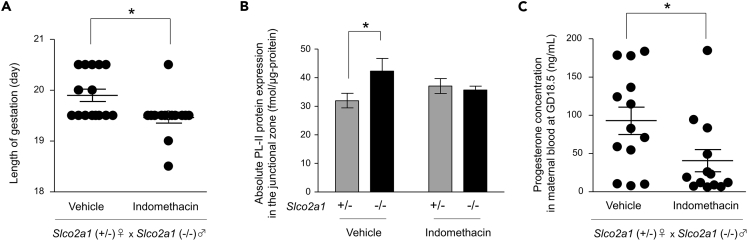
Indomethacin Treatment Rescues the Delayed-Labor Phenotype in *Slco2a1* (+/−) Females Mated with *Slco2a1* (−/−) Males Administration of 1 mg/kg indomethacin on GD15.5 to *Slco2a1* (+/−) females mated with *Slco2a1* (−/−) males. (A) Gestation length of dams treated with vehicle (n = 15) or indomethacin (n = 14). (B) Absolute PL-II expression levels at GD18.5 in the junctional zone of *Slco2a1* (+/−) (n = 7–9) and *Slco2a1* (−/−) placenta (n = 6–10) from *Slco2a1* (+/−) pregnancies treated with vehicle or indomethacin. (C) Circulating progesterone level in maternal blood at GD18.5 from *Slco2a1* (+/−) pregnancies treated with vehicle (n = 13) or indomethacin (n = 13). Data are expressed as the mean ± SEM. ∗p < 0.05, significant difference between groups (A and C, Student's two-tailed t test; B, two-way ANOVA followed by Bonferroni's post hoc test).

In order to address the relationship between high PGE_2_ levels resulting from failure of PGE_2_ degradation at mid-pregnancy and delayed parturition, *Slco2a1* (+/−) females mated with *Slco2a1* (−/−) males were treated with 1 mg/kg indomethacin at GD15.5. This corresponds to the time when degradation of PGE_2_ was inhibited in the junctional zone of *Slco2a1* (−/−) placenta (Figure 3A). *Slco2a1* (+/−) pregnant mice injected with indomethacin delivered significantly earlier than vehicle-treated *Slco2a1* (+/−) pregnant mice (GD19.5 ± 0.1; n = 14 versus GD19.9 ± 0.1; n = 15, p < 0.05) (Figure 6A). Furthermore, we found that the increased expression of PL-II in the junctional zone of *Slco2a1* (−/−) placenta at GD18.5 was blocked by indomethacin (Figure 6B). In accordance with this, indomethacin treatment decreased the circulating progesterone levels (Figure 6C).

## Discussion

In this study, we found that *Slco2a1* (+/−) females mated with *Slco2a1* (−/−) males, giving rise to *Slco2a1* (−/−) and *Slco2a1* (+/−) placentas/fetuses, frequently exhibited delayed parturition (Figure 4A). In contrast, *Slco2a1* (+/−) females mated with wild-type males, giving rise to wild-type and *Slco2a1* (+/−) placentas/fetuses, showed normal delivery (Figure 4A). These results indicate that the high proportion of *Slco2a1* (−/−) conceptuses *in utero* affects the initiation of parturition. In the junctional zone of *Slco2a1* (−/−) placentas, the PL-II level at late pregnancy was significantly increased compared with that in wild-type placentas (Figure 5B). PL-II is suggested to exert luteotropic effects, including suppression of the expression of 20α-HSD*/Akr1c18*, which encodes a progesterone-metabolizing enzyme, in luteal cells (Zhong et al., 1997), resulting in the induction of progesterone release from corpus luteum (Galosy and Talamantes, 1995, Thordarson et al., 1997). In our study, *Akr1c18* expression level in ovary is significantly lower in *Slco2a1* (+/−) females mated with *Slco2a1* (−/−) males than that in the females mated with wild-type males (data not shown), and consistently the circulating progesterone levels were significantly higher in *Slco2a1* (−/−) females mated with *Slco2a1* (−/−) males (Figure 4C). Moreover, the dams with higher progesterone levels at GD18.5 exhibited delayed parturition in crosses of *Slco2a1* (+/−) females to *Slco2a1* (−/−) males (Figure 4D). These findings suggest that delayed progesterone withdrawal and subsequent parturition failure are caused by oversecretion of placenta-derived PL-II in *Slco2a1* (−/−) mice. More than 70% of *Slco2a1* (−/−) pups die soon after birth; therefore, we could not accurately determine the proportion of *Slco2a1* (−/−) pups in the litter, and so the correlation between their proportion and the length of gestation could not be directly determined. However, the contribution of placental PGE_2_ disposition mediated by OATP2A1 to labor induction was further supported by the positive correlation between the proportion of *Slco2a1* (−/−) conceptuses in the litters and maternal progesterone levels at late pregnancy (Figure 4E).

PL-II is abundantly expressed in the junctional zone (Simmons et al., 2008) and PGE_2_ stimulated PL-II production in cultured explants of the junctional zone (Figure 5C). The secretion of PL-II is reportedly stimulated by several factors, such as growth hormone-releasing hormone, dimeric inhibin A, dimeric inhibin B, calcyclin, EGAM1C, and TFAP2C (Kishi et al., 1993, Yamaguchi et al., 1995, Farnsworth and Talamantes, 1998, Saito et al., 2011, Ozturk et al., 2006). Among these factors, PGE_2_ induces dimeric inhibin A production in cultured human granulosa-luteal cells (Eramaa and Ritvos, 1996) and inhibin subunits are expressed in mouse placental junctional zone at the mRNA level (Yamaguchi et al., 1995). Therefore, PGE_2_ may stimulate PL-II production through the induction of dimeric inhibin A expression in the junctional zone. Here, we show for the first time that OATP2A1 is predominantly expressed in the placental junctional zone (Figure 1) and mediates uptake of extracellular PGs by spongiotrophoblasts (Figure 2). Therefore, the defect of PGE_2_ uptake and the subsequent degradation of PGE_2_ by 15-PGDH in the junctional zone of *Slco2a1* (−/−) placenta should increase PL-II expression via an increase of extracellular PGE_2_ in the junctional zone. This possibility is supported by the observation that indomethacin treatment at GD15.5 in *Slco2a1* (+/−) females mated with *Slco2a1* (−/−) males corrected PL-II overproduction in *Slco2a1* (−/−) placenta (Figure 6B). Moreover, indomethacin treatment decreased circulating progesterone levels at GD18.5 (Figure 6C) and corrected the parturition delay (Figure 6A). At GD15.5, COX-1, COX-2, and mPGES-1 are expressed in the placenta, including the junctional zone, although to a much lesser extent (Figures S4B–S4D) (Inagaki et al., 2017). These results raise the possibility that PGE_2_ are synthesized in the junctional zone and/or other placental areas (i.e., labyrinth and decidua). These findings suggest that the uptake of extracellular PGE_2_ by OATP2A1 and subsequent intracellular degradation in the placenta at mid-pregnancy would suppress the signaling for placental PL-II production in late pregnancy, which facilitates progesterone withdrawal for the onset of parturition.

Among fetal tissues, the lung also highly expresses OATP2A1, and OATP2A1 in the lung plays a critical role in the closure of the ductus arteriosus after birth by reducing the level of circulating PGE_2_ (Chang et al., 2010). *Slco2a1* (−/−) neonates die within 24 h after birth because of patent ductus arteriosus, although the labor phenotype was not mentioned in the literature (Chang et al., 2010). Matured fetal lung is reported to be associated with the onset of parturition via secretion of SP-A, which serves as a hormonal signal (Gao et al., 2015). Thus, we have to consider the possibility that OATP2A1 in the fetal lung is involved in the onset of parturition by modulating the SP-A level. However, *Slco2a1* deficiency did not alter the expression level of SP-A in the fetal lung (Figure S5). Accordingly, it seems that OATP2A1 expressed in the fetal lung does not affect SP-A-mediated labor induction signals.

There is considerable evidence that PGs are universal mediators of parturition. At late pregnancy, elevation of PGE_2_ and PGF_2α_ levels in the uterus and ovary promotes uterine myometrial contraction and cervical ripening, which trigger the onset of labor (Romero et al., 2014, Khan et al., 2008, Herington et al., 2018). Since OATP2A1 mediates the uptake of extracellular PGs for intracellular degradation, it is possible that feto-placental *Slco2a1* deficiency elevates PGE_2_ and PGF_2α_ levels even in maternal tissues, including uterus and ovary, and thus causes pre-term delivery. However, in this study, *Slco2a1* (+/−) dams bearing *Slco2a1* (−/−) embryos exhibited delayed parturition (Figure 4A), and feto-placental *Slco2a1* deficiency did not affect the PGE_2_ and PGF_2α_ levels in uterus or ovary at late pregnancy (Figure S6). Therefore, feto-placental OATP2A1 is likely to mediate the reduction of local PG levels in the placenta but may not affect the maternal level. Moreover, we found here that PGE_2_ in the placenta has an opposite signaling function for the maintenance of pregnancy possibly by inducing placental PL-II production (Figures 5 and 6), in contrast to uterine PGE_2_. This difference in the role of PGE_2_ between uterus and placenta was further supported by the results of indomethacin treatment. Indomethacin delays preterm labor because of its tocolytic effect, reducing excessive PG levels in the uterus (Besinger et al., 1991, Morales and Madhav, 1993, Klauser et al., 2014), although there is concern about fetal adverse effects, such as premature closure of the fetal ductus arteriosus (Vermillion et al., 1997, Antonucci et al., 2012). In mice, indomethacin significantly decreased the incidence of preterm labor that was induced by lipopolysaccharide (Lee et al., 2003). On the other hand, in this study, a single administration of indomethacin at mid-pregnancy rescued the delayed-labor phenotype in *Slco2a1* (+/−) females mated with *Slco2a1* (−/−) males (Figure 6A). Our findings provide insight into the role of PGE_2_ in labor induction, indicating that excessive PGE_2_ in the placenta at mid-pregnancy does not facilitate, but rather prevents, the initiation of parturition by modulating endocrine function in the placenta.

In crosses of *Slco2a1* (−/−) mice, 30% of dams exhibited post-term pregnancy, but 30% of dams exhibited pre-term pregnancy (Figure 4A). In this mating, it is necessary to consider the effect of *Slco2a1* deficiency in dams as well as conceptuses. Based on our observations in *Slco2a1* (+/−) females mated with *Slco2a1* (−/−) males, the high proportion of *Slco2a1* (−/−) conceptuses *in utero* causes delayed parturition. Thus, it is reasonable to consider that pre-term parturition is caused primarily by maternal *Slco2a1* deficiency. We confirmed that the uterus expresses OATP2A1 (Figure 1D); therefore, uterine OATP2A1 is likely to be involved in the maintenance of pregnancy by reducing extracellular PG levels in the uterus. Moreover, in *Slco2a1* (−/−) pregnant mice, uterine PG levels might be elevated through an increase in circulating PGs due to the lack of PG degradation in maternal lung. This is consistent with a report showing that, in *Slco2a1* (−/−) male mice, the plasma PGE_2_ level was 3.7-fold higher than in wild-type mice at 1 h after the induction of PGE_2_ by lipopolysaccharide (Nakamura et al., 2018). Moreover, in mice treated with T26A, a specific OATP2A1 inhibitor, for 3 weeks, the plasma PGE_2_ level was 2-fold higher than that in the vehicle control (Chi et al., 2015). Accordingly, maternal *Slco2a1* deficiency is likely to favor preterm delivery owing to the elevated levels of uterine PGs.

At both GD15.5 and GD18.5, [^3^H]PGE_2_ uptake was significantly lower in the junctional zone of *Slco2a1* (−/−) placenta compared with that of wild-type littermates, indicating that OATP2A1 acts as an influx transporter of PGs at GD18.5 as well as GD15.5 (Figure 2). However, the levels of stable metabolites of PGE_2_ and PGF_2α_ in the junctional zone at GD18.5 were similar in both wild-type and *Slco2a1* (−/−) littermates (Figure 3B), although a substantial decrease in PG metabolite levels was observed in *Slco2a1* (−/−) littermates at GD15.5 (Figure 3A). In the case of *Slco2a1* (−/−) mice, the levels of PG metabolites in the junctional zone at GD18.5 were even higher than those at GD15.5, raising the possibility that 15-PGDH and/or 15-oxo-PG Δ^13^-reductase, PG-degrading enzymes, are upregulated to compensate for *Slco2a1* deficiency. In the case of wild-type mice junctional zone, PG metabolite levels at GD18.5 were decreased to approximately half compared with those at GD15.5 (Figure 3), which is consistent with the finding that the placental activity of 15-PGDH at GD18.25 and GD19.0 was decreased from that at GD17.5, becoming similar to that in 15-PGDH hypomorphic mice placenta (Roizen et al., 2008). Accordingly, the uptake of PGs via OATP2A1 at mid-pregnancy is the rate-controlling step for PG degradation, whereas, at late pregnancy, OATP2A1-mediated uptake has little effect on the PG degradation, probably due to the low activity of 15-PGDH.

Based on the observation that, in wild-type mice, placental 15-PGDH activity becomes negligible prior to parturition (Roizen et al., 2008), an increase in extracellular PGE_2_ has the potential to induce PL-II production and subsequent inhibition of progesterone withdrawal. However, prior to parturition, 15-PGDH activity also becomes negligible in the uterus (Roizen et al., 2008), and crossing of 15-PGDH hypomorphic mice resulted in increased levels of PGE_2_ in ovary and PGF_2α_ in uterus and ovary at GD17.5 and GD18.25, leading to pre-term delivery (Roizen et al., 2008). Therefore, it can be considered that, prior to parturition, the luteolytic effect, inducing progesterone withdrawal, of PGF_2α_ in uterus predominates over the luteotropic effect, inducing progesterone production, of PGE_2_ in the placenta through the production of PL-II. On the other hand, in *Slco2a1* (+/−) females mated with *Slco2a1* (−/−) males, owing to overproduction of PL-II, the luteotropic effect of placental PGE_2_ seems to overcome the luteolytic effect of uterine PGF_2α_.

Term labor and preterm labor in humans and rodents are both mediated by elevated levels of PGE_2_ and PGF_2α_ in the uterus, and these PGs induce uterine myometrial contraction and cervical ripening (Herington et al., 2018, Romero et al., 2014). In rodent ovary, PGE_2_ exerts a luteotropic effect, whereas PGF_2α_ is a luteolytic factor (Sugimoto et al., 1997, Henderson et al., 1977, Tamura et al., 2016). In the present study, feto-placental *Slco2a1* deficiency in mice disrupted the timing of parturition by dysregulating the PGE_2_ concentration in the placenta at mid-pregnancy without affecting the PGE_2_ and PGF_2α_ levels in the uterus and ovary at late pregnancy (Figure S6). Whole-exome sequencing has indicated the pathophysiological significance of the *SLCO2A1* gene in human; loss-of-function mutations in *SLCO2A1* are related to primary hypertrophic osteoarthropathy and pachydermoperiostosis (Nakanishi and Tamai, 2017). Although the labor phenotype in humans deficient for OATP2A1 function has not been described so far, *SLCO2A1* is most abundantly expressed in the placenta among human tissues (Kraft et al., 2010). The mechanism of labor induction in humans is considered to be complex; luteolysis does not play a role in human parturition and the circulating progesterone level does not decrease antepartum (Mitchell and Taggart, 2009, Malassine et al., 2003). Nevertheless, it has been reported that, in women with premature uterine contractility, the circulating level of human placental lactogen (hPL) was significantly lower than in women with uncompleted pregnancy between 23 and 28 weeks of gestation (Wojcicka-Jagodzinska et al., 1998), implying that hPL affects the initiation of labor in humans.

In conclusion, we have demonstrated that OATP2A1 is predominantly expressed in spongiotrophoblasts of the junctional zone and is responsible, together with 15-PGDH, for the degradation of PGs in the junctional zone of the placenta at mid-pregnancy. Since PGE_2_ signaling is shown to contribute to the secretion of PL-II, a luteotropic hormone, in the junctional zone, it is possible to consider that PG disposition via OATP2A1 suppresses the secretion of PL-II to induce the production of ovarian progesterone. These results indicate that feto-placental OATP2A1 contributes to the withdrawal of circulating progesterone at late pregnancy, possibly by suppressing placental PL-II production, for the initiation of parturition.

### Limitations of the Study

In our experiments, we demonstrate that the presence of *Slco2a1* (−/−) conceptuses results in delayed parturition of dams by inhibiting progesterone withdrawal and that the expression level of PL-II in *Slco2a1* (−/−) junctional zone is higher than that in the wild-type. PL-II is suggested to exert a luteotropic hormone, but we did not directly demonstrate the causal relationship between the overproduction of PL-II in the junctional zone and delayed parturition. Moreover, we did not clarify the reason why the time lag effect of PGE_2_ signaling on PL-II production occurs. Further experiments will be needed to clarify the mechanism of PL-II production via PGE_2_ signaling and the contribution of PL-II to parturition timing.

### Resource Availability

#### Lead Contact

Further information and requests for resources and reagents should be directed to and will be fulfilled by the Lead Contact, Prof. Masatochi Tomi (tomi-ms@pha.keio.ac.jp).

#### Materials Availability

This study did not generate new unique reagents.

#### Data and Code Availability

The data that support the findings of this study are available from the authors on reasonable request.

## Methods

All methods can be found in the accompanying Transparent Methods supplemental file.

## Supplemental Information

Document S1. Transparent Methods, Figures S1–S6, and Table S1
