# Supplementary material for: Contribution of Prostaglandin Transporter OATP2A1/SLCO2A1 to Placenta-to-Maternal Hormone Signaling and Labor Induction
Source: iScience. 2020 Apr 27;23(5):101098. doi: 10.1016/j.isci.2020.101098 (PMC7225742; doi:10.1016/j.isci.2020.101098)
Supplement: Document S1. Transparent Methods, Figures S1–S6, and Table S1 [file mmc1.pdf]

iScience, Volume *23*

Supplemental Information

Contribution of Prostaglandin Transporter OATP2A1/*SLCO2A1* to Placenta-to-Maternal Hormone Signaling and Labor Induction

Mai Inagaki, Tomohiro Nishimura, Takeo Nakanishi, Hiroaki Shimada, Saki Noguchi, Shin-ichi Akanuma, Masanori Tachikawa, Ken-ichi Hosoya, Ikumi Tamai, Emi Nakashima, and Masatoshi Tomi

Supplemental figures

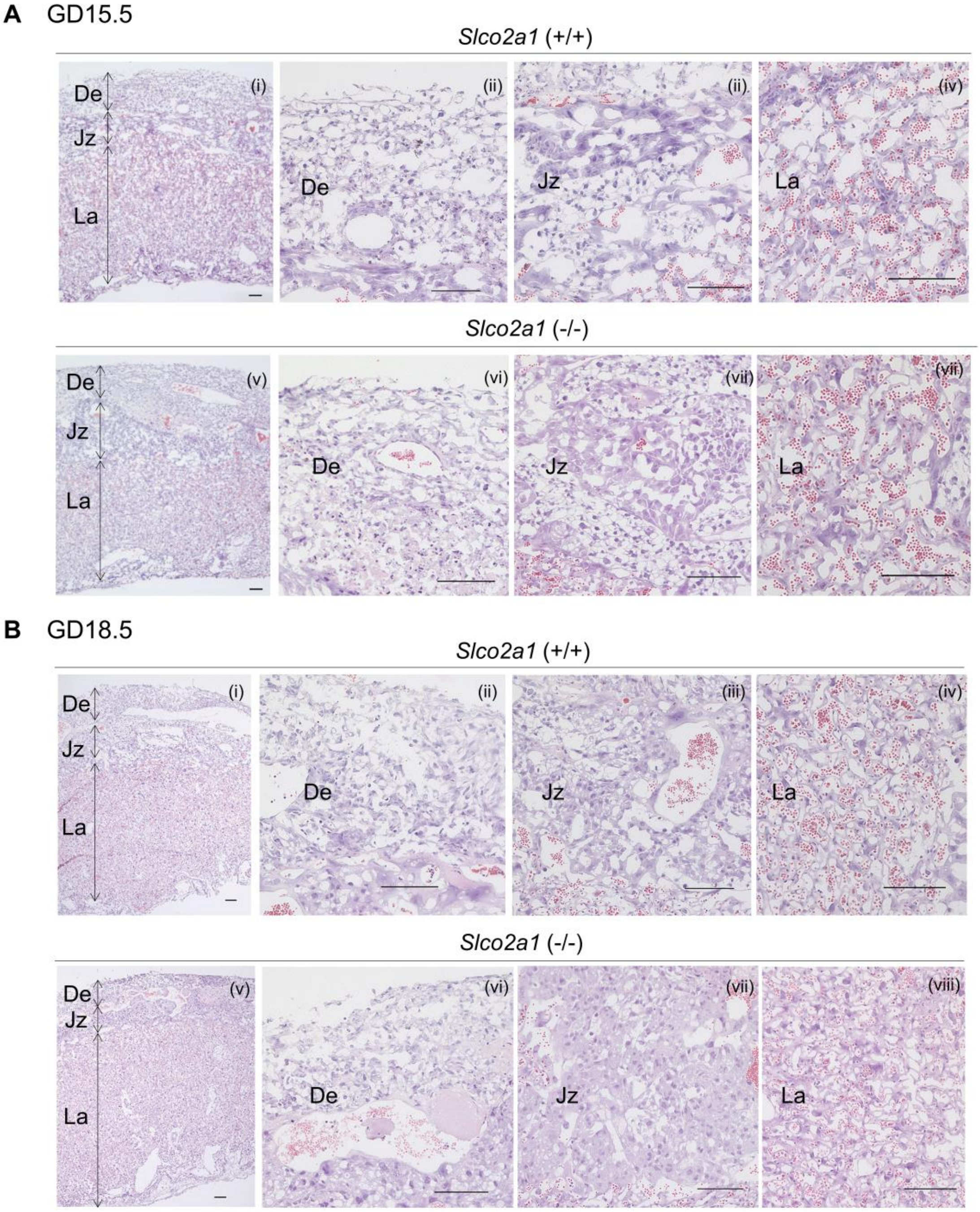

Figure S1: H&E staining of wild-type and *Slco2a1* (-/-) placentas at GD15.5 (A) and GD18.5 (B), Related to Figure 1.

Low (i and v) and high (ii, iii, iv, vi, vii, and viii) magnification. De, decidua; Jz, junctional zone; La, labyrinth. Scale bars = 100 μm.

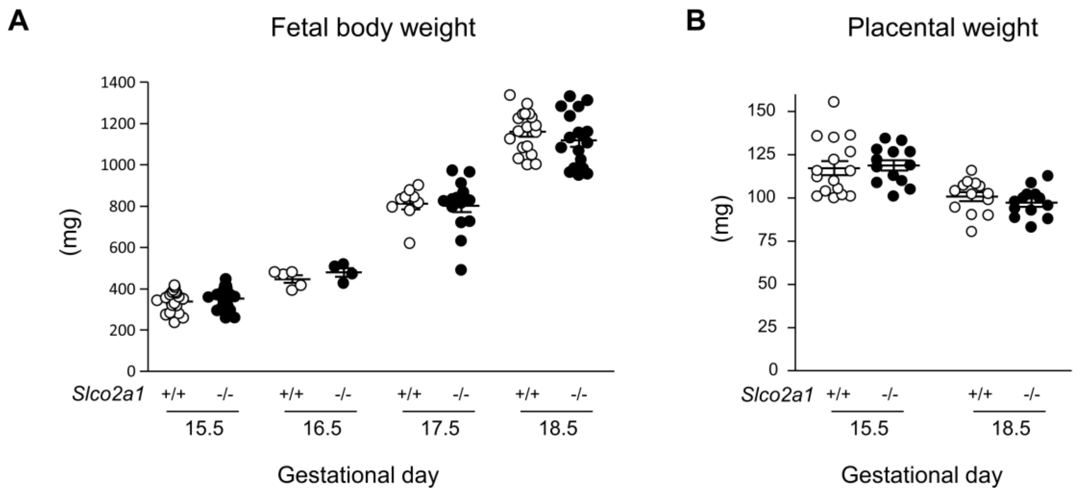

Figure S2: Fetal and placental weights during pregnancy, Related to Figure 1.

(A and B) Weight of wild-type and *Slco2a1* (-/-) fetuses (A) and placentas (B) from GD15.5 to 18.5. Data are expressed as the mean ± SEM (n = 4-22).

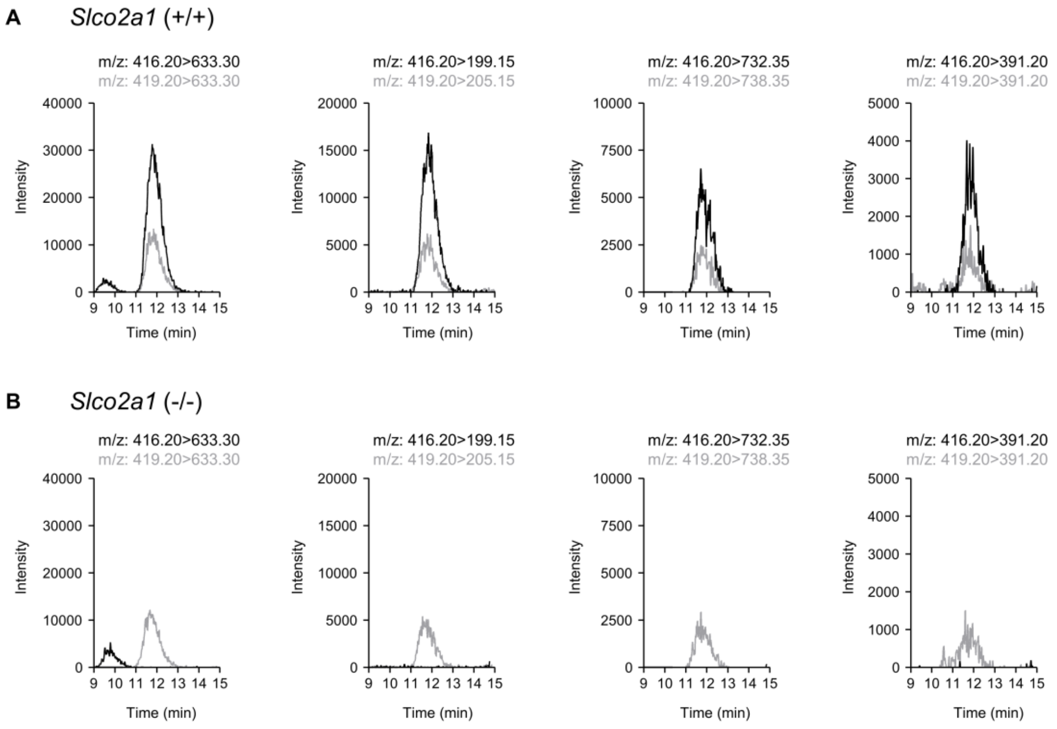

Figure S3: Peak chromatogram of an OATP2A1-specific peptide, VVNQDEK, Related to Figure 1.

Plasma membrane-rich fraction of the placental junctional zone was obtained from wild-type (A) and *Slco2a1* (-/-) (B) pregnancies at GD15.5. Tryptic digests of placental sample spiked with internal standard (IS) peptides were subjected to LC-MS/MS. Peak chromatograms of four SRM transitions for a target peptide (black) and the corresponding IS peptide (gray).

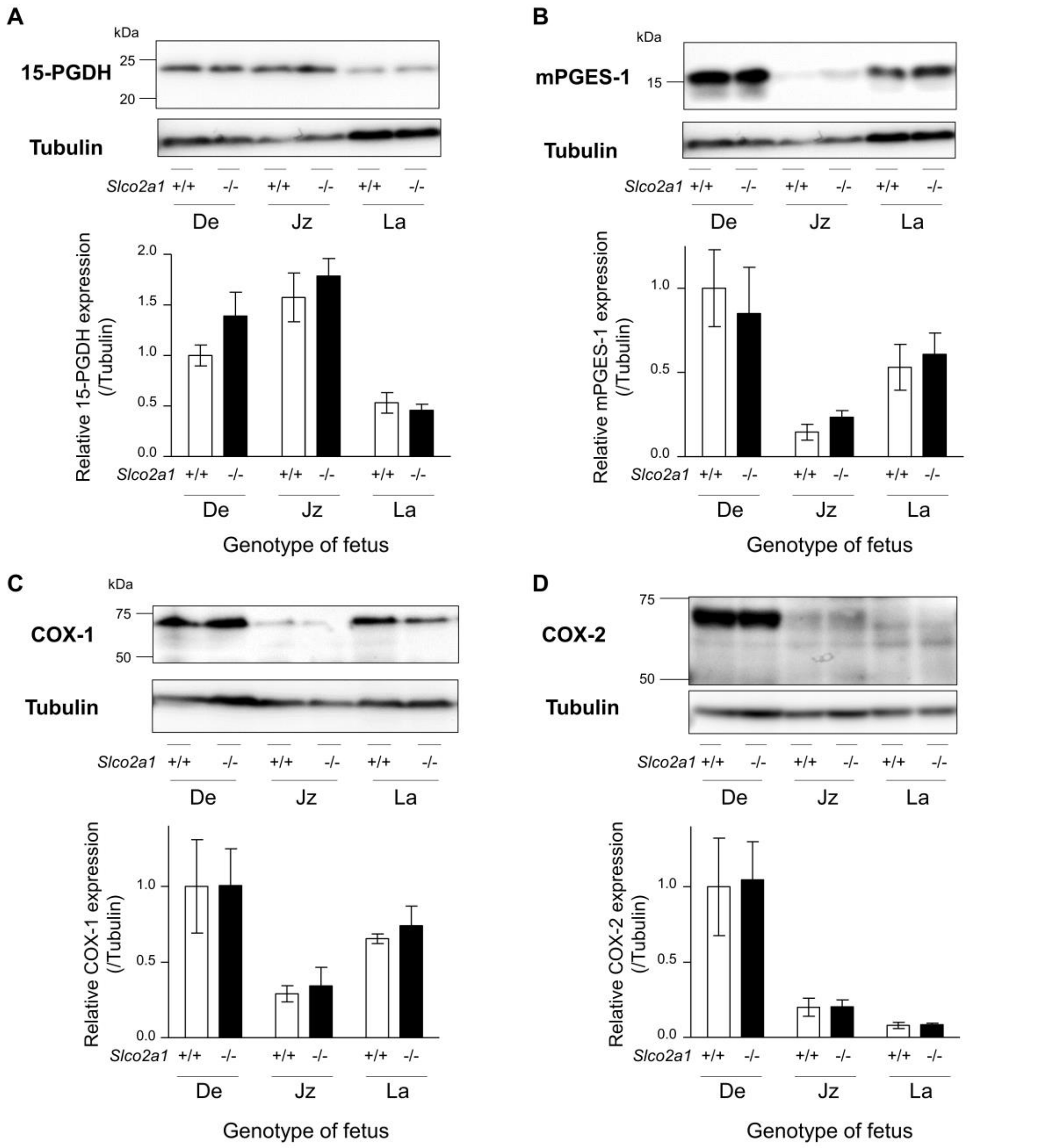

Figure S4: Western blot analysis of 15-PGDH (A), mPGES-1 (B), COX-1 (C), and COX-2 (D) in three placental zones at GD15.5, Related to Figure 3.

Western blot analysis using placental homogenates prepared from wild-type and *Slco2a1* (-/-) mice. Representative images (top) and densitometric analyses of the blots (bottom) from 3 to 4 independent experiments with different placentas are shown. Data are expressed as the mean ± SEM.

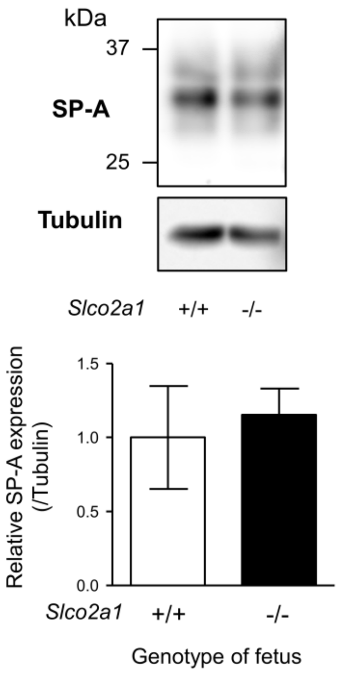

Figure S5: Western blot analysis of surfactant protein A (SP-A) in fetal lung at GD18.5, Related to Figure 4.

Western blot analysis of fetal lung homogenates prepared from wild-type and *Slco2a1* (-/-) mice. Representative images (top) and densitometric analyses of the blots (bottom) from 5 to 7 independent experiments with different placentas are shown. Data are expressed as the mean ± SEM.

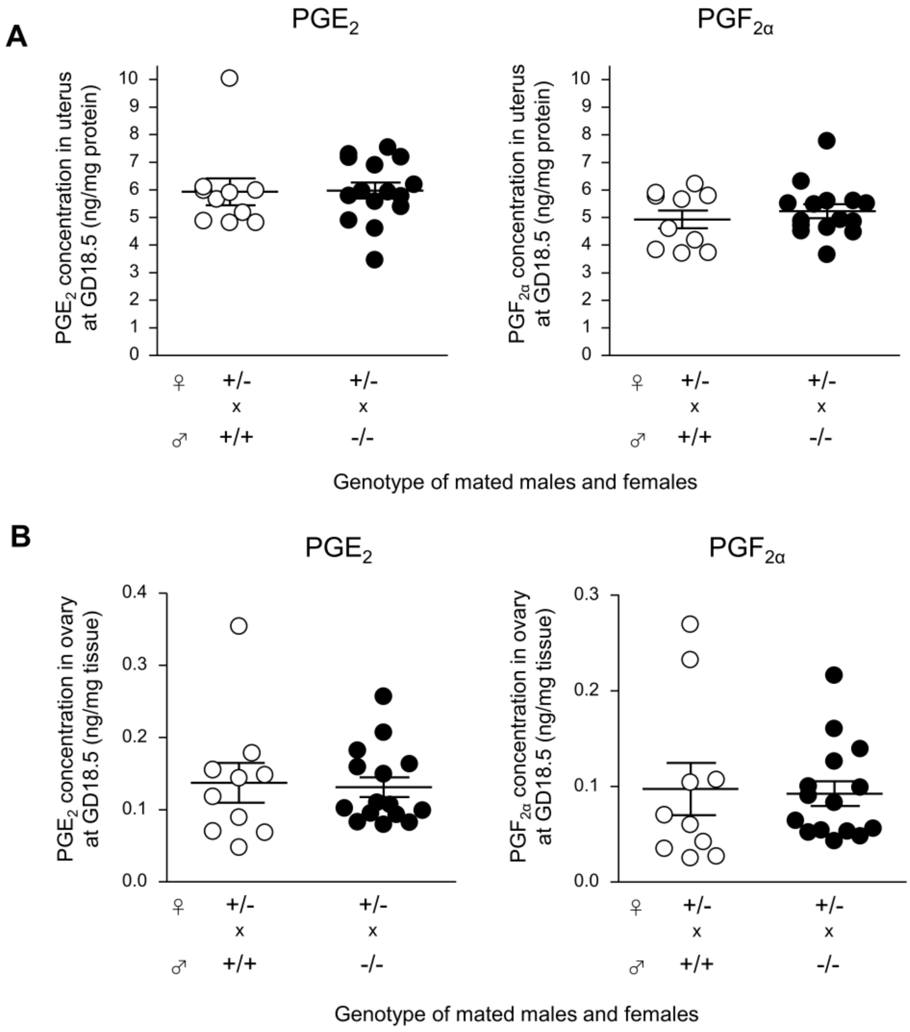

Figure S6: PG levels in ovary, myometrium, and fetal membrane at GD18.5, Related to Figure 4.

PGE_2 and $PGF_{2\alpha}$ levels in uterus (A) and ovary (B) from *Slco2a1* (+/-) females mated with wild-type males (n=10) or *Slco2a1* (-/-) males (n = 15). Data are expressed as the mean ± SEM.

Supplemental table

Gene symbol	ST/IS	Probe sequence	SRM/MRM transition (m/z)				
			Q1	Q3-1	Q3-2	Q3-3	Q3-4
mOATP2A1	ST	VVNQDEK	416.20	633.30	199.15	732.35	391.20
	IS	V**V***NQDEK	419.20	633.30	205.15	738.35	391.20
mPL-I	ST	EFDLDFFDK	588.25	392.15	671.30	556.30	262.15
	IS	EFD**L***DFFDK	591.80	392.15	671.30	556.30	262.15
mPL-II	ST	LPTESLYQR	553.80	211.14	795.40	896.45	666.35
	IS	L**P***TESLYQR	556.80	217.15	795.40	896.45	666.35

Table S1: Target peptide transitions in LC-MS/MS analysis, Related to Figure 1 and 5.

ST and IS mean standard peptides and internal standard peptides, respectively. The stable-isotope-labeled amino acids are indicated with an asterisk.

Transparent Methods

Animals.

All mice were maintained under a 12-hour light/12-hour dark cycle at 25°C with free access to water until use. Animal experiments were approved by the Institutional Animal Care Committee and complied with the standards set out in the Guideline for the Care and Use of Laboratory Animals in Keio University. The presence of a vaginal plug was designated as GD0.5. Parturition was monitored from GD16.5 by observing mice twice daily, in the morning (8:00-9:00 AM) and evening (6:00-8:00 PM). Parturition timing was defined as the observation of the first pup. To ensure that parturition timing was analyzed in mice of the same physiological status, only data on first pregnancies were included.

Slco2a1 (-/-) mice.

To produce *Slco2a1* (flox/+) mice, in vitro fertilization of eggs from C57BL/6 females with sperm cells from *Slco2a1* (flox/flox) males (Nakanishi et al., 2015) was performed and the blastocysts were transferred into pseudopregnant C57BL/6 females. To disrupt the *Slco2a1* gene, *Slco2a1* (flox/+) offspring were interbred with CAG-Cre transgenic mice (RIKEN BRC) (Matsumura et al., 2004), which express Cre recombinase at early stages of development. Mice were genotyped by PCR analysis of genomic DNA from mouse tissue (ear or tail snips) using the forward primers 5'-AGGCTCTCGTGGGGAGTAAT-3' for wild-type mice and 5'-AGGACCTGATAGGCAGCCAA-3' for *Slco2a1* deficient mice, with the reverse primer 5'-CACAGCAGAGACCCAACAGA-3'. Under deep anesthesia with isoflurane, the ovaries, uterus, fetal lungs, and maternal blood were collected from pregnant *Slco2a1* (+/-) female mice mated with wild-type or *Slco2a1* (-/-) males at GD18.5. Blood was collected from the tail vein and plasma was prepared by centrifugation at 10,000 x g for 10 min at 4°C. Placentas were collected at GD15.5 and GD18.5 from pregnant *Slco2a1* (+/-) female mice mated with *Slco2a1* (+/-) male mice. Isolated placentas were dissected into maternal decidua (whitish color), junctional zone (yellowish color), and labyrinth (dark red color) using tweezers (Inagaki et al., 2017). We analyzed qRT-PCR using mRNA gene expression markers (decidua; *Desmin*, junctional zone; *Prl8a8*, Labyrinth; *Syncytin-A*) to validate each layer enrichment.

Histological analysis.

Isolated placentas were fixed overnight in 4% paraformaldehyde in phosphate-buffered saline (PBS) at 4°C and immersed in 20% sucrose before being embedded in OCT compound. Frozen sections were cut at 4 μm and mounted on silanized glass slides. Sections were stained with Mayer's hematoxylin solution (Fujifilm Wako, Osaka, Japan) and Eosin Y (Fujifilm Wako) for

general morphology.

In situ hybridization analyses were performed as described previously (Akashi et al., 2016). Antisense and sense RNA probes for mouse *Slco2a1* (GeneBank accession number NM_03314.4) and *Prl8a8* (NM_001311125.1) mRNAs (nucleotide residues 803-1946 and 56-852, respectively) were labeled with digoxigenin (DIG) or fluorescein-conjugated UTP (Roche, Basel, Switzerland). *Prl8a8* was used as a marker of spongiotrophoblasts (Simmons et al., 2008). Frozen sections were treated with 1 μg/mL proteinase K for 37°C at 30 min, acetylated in 0.25% acetic anhydride/0.1 M triethanolamine for 10 min, and then hybridized overnight with DIG or the fluorescent probe at 60°C. After blocking with blocking regent (Roche), sections were incubated overnight with alkaline phosphate-conjugated anti-DIG antibody (1:2,000) (Roche) or anti-fluorescein antibody (1:2,000) (Roche) at 4°C, and then incubated with nitro-blue tetrazolium chloride and 5-bromo-4-chloro-3-indolyl phosphate at room temperature for color development. Sections were counterstained with Nuclear Fast Red, dehydrated in ethanol, cleared in xylene, and mounted in VectaMount Permanent Mounting Medium (Vector Laboratories).

Frozen sections were incubated with HistoVT One solution (Nacalai Tesque, Kyoto, Japan) for 20 min at 70°C for antigen retrieval. After blocking, sections were incubated with guinea-pig polyclonal OATP2A1 antibody (2 μg/mL) (Tachikawa, Tsuji et al., 2012) and rabbit polyclonal 15-PGDH antibody (1:100) (160615; Cayman Chemical, Ann Arbor, MI) at 4°C overnight, then incubated with species-specific Alexa Fluoro 594-conjugated and Alexa Fluoro 488-conjugated secondary antibodies (Thermo Fisher Scientific, Waltham, MA) at room temperature for 1 hour, and mounted in Vectashield Hard Set Mounting Medium with DAPI (Vector Laboratories, Burlingame, CA).

Immunoblot analysis.

The placentas and fetal lungs were homogenized in cell lysis buffer (Cell Signaling Technology, Danvers, MA). Proteins were separated by SDS–polyacrylamide gel electrophoresis, and electroblotted onto a polyvinylidene difluoride membrane. After blocking, the membrane was incubated at 4°C overnight with rabbit polyclonal anti-15-PGDH antibody (1:2,000), rabbit polyclonal anti-COX-1 antibody (1:500) (160109; Cayman Chemical), rabbit polyclonal anti-COX-2 antibody (1:500) (160126; Cayman Chemical), guinea-pig polyclonal anti-mPGES-1 antibody (0.3 μg/mL) (Tachikawa, Ozeki et al., 2012), rabbit polyclonal anti-SP-A antibody (1:1,000) (ab115791; abcam, Cambridge, UK), or rat monoclonal anti-tubulin antibody (1:5,000) (ab6160; abcam), and subsequently with species-specific horseradish peroxidase-conjugated secondary antibodies. The bands were visualized with ECL Prime Western Blotting Detection reagent (GE Healthcare, Buckinghamshire, UK) and

quantitated by densitometric analysis using a CS Analyzer 2.0 (ATTO, Tokyo, Japan).

[^{3}H]Prostaglandin E_2 uptake.

Explants of each placental zone were pre-incubated with extracellular fluid (ECF) buffer (122 mM NaCl, 25 mM $NaHCO_3$, 3 mM KCl, 1.4 mM $CaCl_2$, 2 mM $MgSO_4$, 0.4 mM K_2HPO_4, 10 mM D-glucose, 10 mM HEPES, pH 7.4) at 37°C for 10 min. After pre-incubation, explants of decidua, junctional zone, and labyrinth were incubated for 20 min in ECF buffer supplemented with 5.6 nM [^{3}H]PGE_2 (prostaglandin E_2 [5,6,8,11,12,14,15-3H(N)]; PerkinElmer, Boston, MA). The uptake was terminated by removal of the buffer and the explants were immediately washed with ice-cold ECF buffer. The explants were homogenized in saline, scintillation cocktail was added, and the radioactivity was measured with a liquid scintillation counter.

Mouse placental explant culture.

Placenta was cut in half, placed in 48-well plates with 400 µL of sterile culture medium (45% DMEM/45% Ham's F12/10% FBS supplemented with penicillin and streptomycin), and incubated at 37°C in a humidified incubator under 20% O_2 and 5% CO_2. After preincubation for 1 hour, all explants were left in culture media containing 0.04% DMSO in the absence or presence of 1 µM PGE_2. The culture medium was changed every 12 hours, and after 24 hours the explants were collected and stored at -80°C until use.

Preparation of plasma membrane-enriched fraction.

Plasma membrane-enriched fractions were prepared as described previously with some modifications (Nishimura et al., 2019). Murine tissues were homogenized in Tris-sucrose buffer (250 mM sucrose, 10 mM Tris-HCl, 1 µM pepstatin A, 10 µM leupeptin, 100 µM phenylmethanesulfonyl fluoride, pH 7.4). The homogenate was centrifuged at 5,800 x g for 15 min at 4°C, and the supernatant was centrifuged again at 10,000 x g for 15 min at 4°C. The resulting supernatant was centrifuged at 100,000 x g for 30 min at 4°C, and the pellet was resuspended in Tris-sucrose buffer. The suspension was overlaid on 38% (w/v) sucrose solution and centrifuged at 100,000 x g for 40 min at 4°C with a swing-out rotor. The turbid layer at the interface was collected and diluted with 10 mM Tris-HCl buffer (pH 7.4). The resulting suspension was centrifuged at 100,000 x g for 40 min at 4°C to afford the plasma membrane-enriched fraction as a pellet.

LC-MS/MS-based targeted protein quantification analysis.

Absolute protein expression levels were determined by quantifying the absolute amounts of specific peptides produced by trypsin digestion of OATP2A1 and PLs using LC-MS/MS as

described previously, with some modifications (Nishimura et al., 2019, Uchida et al., 2013). Fifty micrograms of protein was reduced with dithiothreitol (Fujifilm Wako) and S-carbamoylmethylated with iodoacetamide (Fujifilm Wako). The S-carbamoylmethylated samples were precipitated with a mixture of methanol and chloroform. The precipitates were dissolved in 6 M urea, diluted 5-fold with 100 mM Tris-HCl (pH 8.5), and treated at room temperature for 3 hours with proteaseMAX^{TM} surfactant (Promega, Madison, WI) and lysyl endopeptidase (Wako Pure Chemical Industries) at a final concentration of 0.05% and a final enzyme/substrate ratio of 1:100, respectively. The samples were then digested with sequence-grade modified trypsin (Promega) at an enzyme/substrate ratio 1:100 at 37°C for 16 hours. The resulting peptides were spiked with internal standard peptides, which are stable isotope (^{13}C and ^{15}N)-labeled peptides having the same amino acid sequences as the analyte peptides, and acidified with formic acid.

The LC-MS/MS system consisted of an HPLC instrument (Shimadzu, Kyoto, Japan) and an electrospray ionization triple quadrupole mass spectrometer (LCMS-8050, Shimadzu). Mobile phases A and B consisted of 0.1% formic acid in water and 0.1% formic acid in acetonitrile, respectively. Chromatographic separation was performed on an XBridge Peptide BEH C18 column (3.5 μm, 1 mm × 150 mm, Waters, Milford, MA) at 40°C with a linear gradient of mobile phase B as follows: 1% for 0-5 min, 1% to 50% for 5-25 min, 100% for 25-30 min, and 1% for 30-60 min. MS spectrometric detection was performed by multiple reaction monitoring in the electrospray ionization mode. Peak data were extracted by using 4 sets of SRM transitions (m/z) of the precursor ion (Q1) and the product ion (Q3) per peptide, as listed in Supplemental Table 1, with the dwell time of 10 msec per transition. The amount of the peptide in the sample was determined for each transition using the peak area ratio (analyte peptide/corresponding internal standard peptide) of the positive peak and a calibration curve obtained by using known concentrations of synthetic peptides, and expressed as the average of 4 positive peaks from different transitions.

Measurement of prostaglandins and progesterone.

Murine tissues were homogenized with a sonicator in the presence of d4-PGE_2 (Cayman Chemical) as an internal standard. The homogenates were deproteinized with the same volume of methanol. The supernatant was obtained by centrifugation at 15,000 rpm for 5 min at 4°C and applied to a hydrophilized C18 solid-phase extraction cartridge. The eluate was evaporated in a vacuum centrifuge and the pellets were reconstituted in 50% acetonitrile.

The amounts of prostaglandins and progesterone extracted from tissue homogenates were determined by LC-MS/MS. Mobile phases A and B consisted of 0.1% formic acid in water and 0.1% formic acid in acetonitrile, respectively. Chromatographic separation was performed on a

Capcell Pak C18 UG120 column (5 μm, 2.0 mm × 150 mm, Shiseido, Tokyo, Japan) at 40°C with gradients of mobile phase B as follows: 10% to 25% for 0-5 min, 25% to 35% for 5-10 min, 35% to 75% for 10-20 min, 100% for 20-25 min, and 10% for 25-30 min for measurement of prostaglandins; 30% for 0-5 min, 30% to 55% for 5-20 min, 100% for 20-25 min, and 30% for 25-30 min for measurement of progesterone. Mass spectrometric detection was performed by multiple reaction monitoring in the electrospray ionization mode, using m/z 351.50→271.40 for PGE_2, 351.50→175.25 for 13,14-dihydro 15-keto PGE_2, 333.50→175.20 for 13,14-dihydro 15-keto PGA_2, 353.50→193.50 for $PGF_{2\alpha}$, 353.50→113.10 for 13,14-dihydro 15-keto $PGF_{2\alpha}$, 355.50→275.10 for d4-PGE_2, and 315.25→96.95 for progesterone.

RU486 and indomethacin treatment.

The progesterone receptor antagonist RU486 (mifepristone, Wako Pure Chemical Industries) was administered by subcutaneous injection (150 μg in ethanol/mineral oil) to pregnant mice at 4:00 PM on GD18.5, following the method used in a previous study (Dudley et al., 1996). Indomethacin was administered by intraperitoneal injection (1 mg/kg animal in PBS) to pregnant mice at 10:00 AM on GD15.5. Control mice received the vehicle.

Statistics.

Results are presented as mean ± S.E.M. Statistical analysis was performed by means of one-way ANOVA or two-way ANOVA followed by Bonferroni's post hoc test for multiple comparisons or Student's 2-tailed *t* test for comparison between two groups. Linear regression analysis was performed using GraphPad Prism4 (GraphPad Software, San Diego, CA). *P* values of less than 0.05 were considered statistically significant.

Supplemental References

Akashi, T., Nishimura, T., Takaki, Y., Takahashi, M., Shin, B.C., Tomi, M., and Nakashima, E. (2016). Layer II of placental syncytiotrophoblasts expresses MDR1 and BCRP at the apical membrane in rodents. Reprod.Toxicol. *65,* 375-381.

Dudley, D.J., Branch, D.W., Edwin, S.S., and Mitchell, M.D. (1996). Induction of preterm birth in mice by RU486. Biol.Reprod. *55,* 992-995.

Inagaki, M., Nishimura, T., Akanuma, S.I., Nakanishi, T., Tachikawa, M., Tamai, I., Hosoya, K.I., Nakashima, E., and Tomi, M. (2017). Co-localization of microsomal prostaglandin E synthase-1 with cyclooxygenase-1 in layer II of murine placental syncytiotrophoblasts. Placenta. *53,* 76-82.

Matsumura, H., Hasuwa, H., Inoue, N., Ikawa, M., and Okabe, M. (2004). Lineage-specific cell disruption in living mice by Cre-mediated expression of diphtheria toxin A chain. Biochem.Biophys.Res.Commun. *321,* 275-279.

Nakanishi, T., Hasegawa, Y., Mimura, R., Wakayama, T., Uetoko, Y., Komori, H., Akanuma, S., Hosoya, K., and Tamai, I. (2015). Prostaglandin Transporter (PGT/SLCO2A1) Protects the Lung from Bleomycin-Induced Fibrosis. PLoS One. *10,* e0123895.

Nishimura, T., Sano, Y., Takahashi, Y., Noguchi, S., Uchida, Y., Takagi, A., Tanaka, T., Katakura, S., Nakashima, E., Tachikawa, M., Maruyama, T., Terasaki, T., and Tomi, M. (2019). Quantification of ENT1 and ENT2 Proteins at the Placental Barrier and Contribution of These Transporters to Ribavirin Uptake. J.Pharm.Sci.

Simmons, D.G., Rawn, S., Davies, A., Hughes, M., and Cross, J.C. (2008). Spatial and temporal expression of the 23 murine Prolactin/Placental Lactogen-related genes is not associated with their position in the locus. BMC Genomics. *9,* 352-2164-9-352.

Tachikawa, M., Ozeki, G., Higuchi, T., Akanuma, S., Tsuji, K., and Hosoya, K. (2012). Role of the blood-cerebrospinal fluid barrier transporter as a cerebral clearance system for prostaglandin E(2) produced in the brain. J.Neurochem. *123,* 750-760.

Tachikawa, M., Tsuji, K., Yokoyama, R., Higuchi, T., Ozeki, G., Yashiki, A., Akanuma, S., Hayashi, K., Nishiura, A., and Hosoya, K. (2012). A clearance system for prostaglandin D2, a

sleep-promoting factor, in cerebrospinal fluid: role of the blood-cerebrospinal barrier transporters. J.Pharmacol.Exp.Ther. *343,* 608-616.

Uchida, Y., Tachikawa, M., Obuchi, W., Hoshi, Y., Tomioka, Y., Ohtsuki, S., and Terasaki, T. (2013). A study protocol for quantitative targeted absolute proteomics (QTAP) by LC-MS/MS: application for inter-strain differences in protein expression levels of transporters, receptors, claudin-5, and marker proteins at the blood-brain barrier in ddY, FVB, and C57BL/6J mice. Fluids Barriers CNS. *10,* 21-8118-10-21.
